# Real life results in using 5-ASA for maintaining mild to moderate UC patients in Japan, a multi-center study, OPTIMUM Study

**DOI:** 10.1186/s12876-017-0604-y

**Published:** 2017-04-04

**Authors:** Masakazu Nagahori, Shuji Kochi, Hiroyuki Hanai, Takayuki Yamamoto, Shiro Nakamura, Soji Omuro, Mamoru Watanabe, Toshifumi Hibi, OPTIMUM Study Group

**Affiliations:** 1 Department of Gastroenterology, Tokyo Medical and Dental University, 1-5-45, Yushima, Bunkyoku, Tokyo, 113-8519 Japan; 2 Division of Gastroenterology, Matsuyama Red Cross Hospital, 1 Bunkyocho, Matsuyama, Ehime Japan; 3 Center for Gastroenterology and Inflammatory Bowel Disease Research, Hamamatsu South Hospital, 26 Shirowacho, Minamiku, Hamamatsu, Shizuoka Japan; 4 Inflammatory Bowel Disease Center, Yokkaichi Hazu Medical Center, 10-8 Hazuyamacho, Yokkaichi, Mie Japan; 5 Department of Inflammatory Bowel Disease, Division of Internal Medicine, Hyogo College of Medicine, 1-1 Mukogawacho, Nishinomiya, Hyogo Japan; 6 Medical Affairs, Clinical Development Center, Kyorin Pharmaceutical Co., Ltd. 4-6 Kanda Surugadai, Chiyodaku, Tokyo, Japan; 7 Center for Advanced IBD Research and Treatment, Kitasato Institute Hospital, Kitasato University, 5-9-1 Shirokane, Minatoku, Tokyo, Japan

**Keywords:** Ulcerative colitis, Observational study, Oral 5-ASA products, Remission maintenance rate

## Abstract

**Background:**

Efficacy of maintenance therapy in ulcerative colitis (UC) in the remission stage has been reported to depend on release profile or dosing regimen of oral 5-aminosalicylic acid (5-ASA) products used. Aim of this study is to investigate real life results in using oral 5-ASA products for maintaining mild to moderate UC patients in Japan.

**Methods:**

Adult UC outpatients treated with oral 5-ASA products were enrolled from 379 sites in Japan between July 2012 and July 2013, and followed for 52 weeks. Remission maintenance rate was evaluated by products and dosages. Factors affecting recurrence were also examined.

**Results:**

A total of 5695 UC patients were registered. Among the 4677 patients in whom remission maintenance was observed, remission maintenance rate at week 52 was 80.2%. As for disease duration and dosage, Pentasa® 4000 mg/day in 2 divided doses was administered to 480 (21.0%) patients in remission and 341 (46.6%) patients in active stage, and Asacol® 3600 mg/day in 3 divided doses was administered to 696 (46.4%) patients in remission and 473 (67.3%) patients in active stage.

The remission maintenance rate at week 52 by dosage and frequency did not significantly differ between Pentasa® Tablets at 4000 mg/day in 2 divided doses (76.5%) and Asacol® Tablets at 3600 mg/day in 3 divided doses (76.1%, *P* = 0.7868).

Factors affecting the risk of relapse in UC were identified. Significantly persistent remission maintenance was noted in patients in whom duration of remission maintenance until enrollment was 12 to <24 months or ≥24 months relative to the reference category of <3 months (12 to <24 months: HR 0.600 [0.486–0.740], *p* < 0.0001]; ≥24 months: HR 0.352 [0.289–0.431], *p* < 0.0001).

**Conclusions:**

Efficacy of real life results in using oral 5-ASA products for maintaining mild to moderate UC patients was favorable. Maintaining remission for 12 months or longer after induction therapy was shown to reduce recurrence risk thereafter.

**Trial registration:**

UMIN 000008563 (the date of registration: July 30, 2012), ClinicalTrials.gov NCT01654783 (the date of registration: July 30, 2012)

**Electronic supplementary material:**

The online version of this article (doi:10.1186/s12876-017-0604-y) contains supplementary material, which is available to authorized users.

## Background

Ulcerative colitis (UC) is a diffuse non-specific inflammatory bowel disease of unknown cause, characterized by erosions and ulcers in the large intestine. Since designation in 1973 as a specified disease (intractable disease) by the Ministry of Health and Welfare (at that time) in Japan, the patient population has been growing and exceeded 160 thousand patients in 2013, as estimated based on the number of medical care certificates and registration certificates issued [1].

The medical therapy for UC involves remission induction in the active stage and remission maintenance after induction has been achieved. The Guidelines for Treatment of Ulcerative Colitis 2014 (internal medicine) [2] recommend remission maintenance with 5-aminosalycilic acid (5-ASA) products (oral agent, enema, suppository), as well as immunomodulators (azathioprine, 6-MP), infliximab infusion, and adalimumab subcutaneous injection. Among these drugs, 6-MP has not been approved UC in Japan. For infliximab and adalimumab, scheduled maintenance therapy is recommended only for cases that achieved remission induction with the drug.

In Japan, the following 5-ASA products are approved and widely used as first-line drugs for treatment of mild to moderate UC: Salazopyrin® (salazosulfapyridine) tablets, approved in 1969; Salazopyrin® suppositories, in 1981; Pentasa® tablets, 1996; Pentasa® enema, 2002; Asacol® tablets, 2009; Pentasa® suppositories, 2013; and Pentasa® granules, approved in 2015. After remission induction has been achieved, 5-ASA products are continued for remission maintenance. 5-ASA products are superior to placebo for maintenance therapy [3]. Regarding doses of 5-ASA products in remission maintenance, one study has reported that continued use of the same dose as used for remission induction led to long-term remission maintenance [4], while another has reported that remission maintenance rate and patient satisfaction with prescription differed depending on the dosage and frequency [5].

Aim of this study is to investigate real life results in using oral 5-ASA products for maintaining mild to moderate UC patients in Japan. In addition, risk factors for relapse during the observation period were also investigated.

## Methods

### Study population

Among UC outpatients who visited 379 medical institutions in Japan between July 2012 and July 2013, those diagnosed with mild to moderate active-stage UC or remission-stage UC based on the Diagnostic Criteria in Ulcerative Colitis (revised on February 13, 2010) [6] and receiving remission induction therapy or remission maintenance therapy with oral 5-ASA products (Pentasa® Tablets, Asacol® Tablets, Salazopyrin® Tablets, and generics of these drugs) were included in the study. The patients were enrolled in sequential order at site visit. The following patients were excluded from the study: severe/fulminant active-stage UC patients, patients undergoing total/subtotal proctocolectomy, patients complicated with a malignancy, pregnant or potentially pregnant patients; and others judged ineligible by the investigator or subinvestigators.

### Patient observation and evaluation

The efficacy for remission maintenance was evaluated during the observation period, at enrollment, week 26, and week 52. Patients observed for longer than 52 weeks were included in the analysis of evaluation at week 52.

At enrollment, patients were examined for the following demographics: age, sex, height, weight, month and year of diagnosis, classification of disease type based on the extent of lesion (total colitis, left-sided colitis, proctitis, others), classification by clinical course (first onset type, relapse-remitting type, chronic continuous type), smoking habit, drinking habit, employment status, and complications. Clinical course was classified as follows: one attack only; relapse-remitting type, in which the relapse and the remission are repeated; chronic continuous type, in which the symptoms persist for >6 months from the onset. Drinking habit was classified as follows; No habit; once a week and less, Habit; twice a week and more. Smoking habit was classified as follows: No habit; non-smoker, Habit; smoker and ex-smoker. The complications were intended for all diseases. Patients diagnosed with UC in the remission stage at enrollment were also examined for the duration of remission maintenance until enrollment.

At enrollment, week 26, and week 52, patients were evaluated using partial UCDAI (pUCDAI) consisting of stool frequency, bloody stool, and physician’s global assessment (PGA), which was designed using the UC disease activity index (UCDAI) by Sutherland, et al. [7] as reference. This was a 4-point scale from 0 to 3 (maximum total: 9), with higher score indicating severer symptom. The results of endoscopy, if performed, were also evaluated on a 4-point scale. The pUCDAI was reported on the case report form. Disease stage (remission, relapse) was judged by attending UC specialist.

If prescription of an oral 5-ASA product was changed during the observation period, the name, dose, and frequency of the drug as well as the reason for the change were recorded. If the disease stage or prescription of an oral 5-ASA product was changed, changes during the observation period from enrollment through week 26 were documented in the evaluation at week 26, and those during the observation period after week 26 were documented in the evaluation at week 52, based on the data in the patient’s medical record. The adherence to medication of oral 5-ASA products was investigated using a visual analogue scale (VAS) of a questionnaire on adherence to medication of oral 5-ASA products (Additional file 1). Aim of this study is to investigate real life results in using oral 5-ASA products for maintaining mild to moderate UC patients in Japan. Because We did not acquire the safety data.

### Study endpoints

The primary endpoint in the present study was the percentage of patients who maintained remission throughout the observation period until week 52 (remission maintenance rate). Remission maintenance rate was calculated by totaling the percentage of UC patients in the remission stage at enrollment who maintained remission throughout the observation period and the percentage of UC patients in the active stage at enrollment who achieved remission induction by remission induction therapy and maintained remission for the rest of the observation period.

The secondary endpoints were the number of relapses (the number of relapses per year from enrollment of remission-stage cases or from the first remission introduction after enrollment of active-stage cases, as determined by the person-years method), duration of remission maintenance (the number of days from enrollment of remission-stage cases or from remission induction of active cases until the first relapse or discontinuation), and drug adherence rate.

UC patients who were in the active stage at enrollment or who were in the remission stage at enrollment but experienced relapse thereafter were examined for post-induction dose of oral 5-ASA products to calculate remission maintenance rate separately for patients who received the same dose as used in remission induction or patients who received a reduced dose. In addition, risk factors for relapse were also investigated during the observation period.

### Statistical analysis

Ito, et al. have reported 1-year remission maintenance of 76 to 80% with oral 5-ASA products [8]. In the present study, which was an observational study, 1-year remission maintenance rate with oral 5-ASA products was assumed to be 80%. The necessary sample size to provide at least 80% power to detect 5% difference in remission maintenance rate as statistically significant difference at a significance level of α = 0.05 was calculated to be at least 906 patients per group, or at least 1812 patients for 2 groups. As the Guidelines for Treatment of Ulcerative Colitis recommend various dosing regimens of oral 5-ASA products in remission maintenance for UC patients, it is expected that various dosing regimens are actually used in clinical practice. Therefore, assuming that 1812 patients in the 2 largest study groups account for approximately 2/5 (40%) of the overall study population, enrollment of 4530 patients was considered necessary. Although they were expected to visit study sites regularly, the target sample size was set at 5000, allowing for a dropout rate of approximately 10%. Patient baseline characteristics were expressed as actual number and percentage for categorical variables and as mean ± standard deviation (SD) for continuous variables. The data of the endpoints were expressed as mean (±SD). Comparison of remission maintenance rate by drug group was performed using Cochran-Mantel-Haenszel test [9, 10] for the following adjustment factors: age (≤60 years vs. ≥61 years), classification by the extent of lesion, classification by clinical course, smoking habit, concomitant use of drugs for UC other than probiotics/cytapheresis therapy, duration of disease, and duration of remission maintenance until enrollment (0 for patients in the active stage at enrollment). Note that analyses by the extent of lesion and by clinical course were conducted after exclusion of classification by the extent of lesion and classification by clinical course, respectively, from adjustment factors. To identify factors affecting relapse, the following items were analyzed by stepwise selection in the Cox regression model: age, sex, BMI, duration of disease, classification by the extent of lesion, classification by clinical course, smoking habit, drinking habit, complications, concomitant use of infliximab or adalimumab, concomitant use of tacrolimus, concomitant use of an immunomodulator, concomitant use of probiotics, concomitant use of a 5-ASA enema or suppository, drug adherence at enrollment, type of oral 5-ASA products used, daily dose of oral 5-ASA products, duration of remission maintenance until enrollment (0 for patients in the active stage at enrollment), and stool frequency score, bloody stool score, and PGA score at enrollment.

## Results

### Enrolled patients

**Fig. 1:**
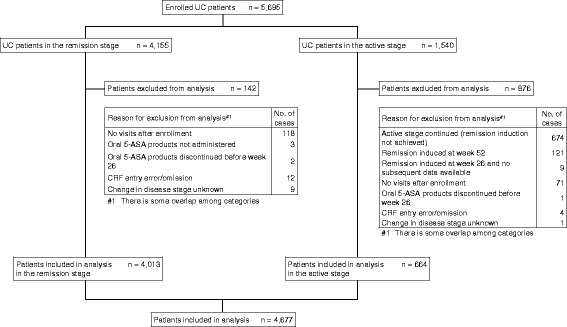
Patient Disposition

A total of 5695 UC patients were enrolled from 379 sites in Japan. Of these, 4155 patients were in the remission stage, and 1540 patients were in the active stage. Of all patients enrolled, 4677 patients were included in the observational study of remission maintenance, and 1018 patients were excluded (Fig. 1).

### Baseline characteristics

The patient baseline characteristics of 5695 patients enrolled and 4677 patients included in the observational study of remission maintenance are shown in Table 1. Among the 5695 patients enrolled, oral 5-ASA products used was Pentasa® Tablets in 3023 patients (53.1%), Asacol® Tablets in 2202 patients (38.7%), Salazopyrin® Tablets in 419 patients (7.4%), a generic of any of these drugs in 20 patients (0.4%), combination of oral 5-ASA drugs in 28 patients (0.5%), and not indicated in 3 patients (0.1%). Daily dose of each drug was 250 mg/day to 6000 mg/day for Pentasa® Tablets, 400 mg/day to 4800 mg/day for Asacol® Tablets, and 500 mg/day to 8000 mg/day for Salazopyrin® Tablets.

**Table 1:** Patient Baseline Characteristics

Item			No. of enrolled patients (*n* = 5, 695)^a^		No. of patients receiving remission maintenance (*n* = 4, 677)^b^	
			No. of patients	(%)	No. of patients	(%)
Sex	Male		3188	(56.0)	2610	(55.8)
	Female		2505	(44.0)	2065	(44.2)
	Unknown		2	(0.0)	2	(0.0)
Age (years)	<20		8	(0.1)	4	(0.1)
	20–30		713	(12.5)	538	(11.5)
	31–40		1327	(23.3)	1063	(22.7)
	41–50		1371	(24.1)	1114	(23.8)
	51–60		1010	(17.7)	869	(18.6)
	61 - 70		826	(14.5)	713	(15.2)
	≥71		435	(7.6)	373	(8.0)
	Not indicated		5	(0.1)	3	(0.1)
BMI	<18.5		609	(10.7)	485	(10.4)
	18.5–<25.0		4061	(71.3)	3354	(71.7)
	≥25.0		975	(17.1)	801	(17.1)
	Not indicated		50	(0.9)	37	(0.8)
Disease stage	Disease stage		No. of patients	(%)	No. of patients	(%)
	Active stage	Mild	1303	(22.9)	574	(12.3)
		Moderate	236	(4.1)	89	(1.9)
		Not indicated	1	(0.0)	1	(0.0)
	Remission stage		4155	(73.0)	4013	(85.8)
Extent of lesion	Total colitis type		2378	(41.8)	1942	(41.5)
	Left-sided colitis type		1951	(34.3)	1391	(34.0)
	Proctitis type		1243	(21.8)	1043	(22.3)
	Other		121	(2.1)	99	(2.1)
	Not indicated		2	(0.0)	2	(0.0)
Clinical course	First onset type		953	(16.7)	838	(17.9)
	Relapse-remitting type		3995	(70.1)	3427	(73.3)
	Chronic continuous type		745	(13.1)	410	(8.8)
	Not indicated		2	(0.0)	2	(0.0)
Drinking habit	No		3250	(57.1)	2631	(56.3)
	≤1 day/week		1201	(21.1)	992	(21.2)
	2 - 5 days/week		712	(12.5)	595	(12.7)
	≥6 days/week		513	(9.0)	443	(9.5)
	Not indicated		19	(0.3)	16	(0.3)
Smoking habit	No		4207	(73.9)	3442	(73.6)
	Yes		1473	(25.9)	1222	(26.1)
	Not indicated		15	(0.3)	13	(0.3)
Complications	Yes		1166	(20.5)	966	(20.7)
	No		4529	(79.5)	3711	(79.3)
Employment status	Student		136	(2.4)	108	(2.3)
	Part-time worker		645	(11.3)	534	(11.4)
	Full-time worker		3242	(56.9)	2632	(56.3)
	Other		1666	(29.3)	1401	(30.0)
	Not indicated		6	(0.1)	1	(0.0)
Use of oral 5-ASA agent	Pentasa®		3023	(53.1)	2528	(54.1)
	Asacol®		2202	(38.7)	1743	(37.3)
	Salazopyrin®		419	(7.4)	368	(7.9)
	Other (generic)		20	(0.4)	16	(0.3)
	Concomitant use (oral products)		28	(0.5)	22	(0.5)
	Not indicated		3	(0.1)	0	(0.0)
concomitant medication	Yes		2669	(46.9)	2076	(44.4)
	No		3026	(53.1)	2601	(55.6)
	probiotics		960	(16.9)	789	(16.9)
	topical 5-ASA		810	(14.2)	593	(12.7)
	topical steroid		259	(4.5)	170	(3.6)
	systemic steroid		401	(7.0)	275	(5.9)
	immunomodulator		870	(15.3)	699	(14.9)
	anti-TNF		169	(3.0)	129	(2.8)
	tacrolims		52	(0.9)	26	(0.6)
	cyclosporine		1	(0.0)	0	(0.0)
	leukocyte apheresis		37	(0.6)	21	(0.4)
pUCDAI and score of each category (Mean ± SD)	pUCDAI		0.9 ± 1.5 (*n* = 5694)		0.6 ± 1.2 (*n* = 4677)	
	Stool frequency		0.4 ± 0.7 (*n* = 5694)		0.3 ± 0.6 (*n* = 4677)	
	Bloody stool		0.2 ± 0.5 (*n* = 5695)		0.1 ± 0.4 (*n* = 4677)	
	PGA		0.3 ± 0.5 (*n* = 5695)		0.2 ± 0.4 (*n* = 4677)	
Duration of disease (mean ± SD) (months)			108.2 ± 93.5		110.5 ± 95.0	
			(*n* = 5670)		(*n* = 4658)	
Duration of remission maintenance until enrollment			31.4 ± 40.3		31.6 ± 40.5	
(mean ± SD) (months)			(*n* = 4107)		(*n* = 3958)	
Drug adherence (mean ± SD)			90.5 ± 14.2% (*n* = 5648)		90.7 ± 13.7% (*n* = 4646)	

^a^%: Percentage of a total of 5695 patients

^b^Subjects other than those remaining in the active stage throughout the observation period from enrollment. %: Percentage of a total of 4677 patients

**Fig. 2:**
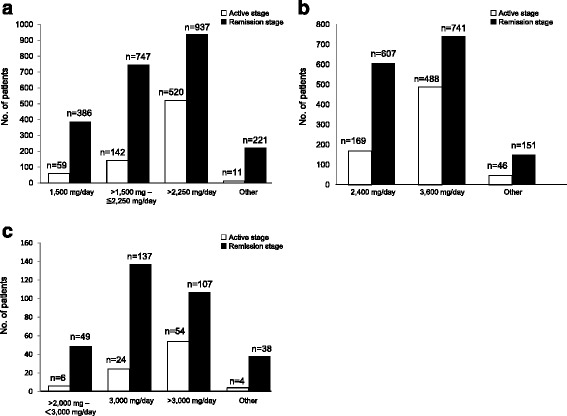
Number of Patients by Disease Stage at Enrollment and by Drug. **a** Daily dose of Pentasa® **b** Daily dose of Asacol® **c** Daily dose of Salazopyrin®

By stage/dosage, the most frequently used dose of Pentasa® Tablets was >2250 mg/day in both remission stage and active stage (937 patients in the remission stage, 520 patients in the active stage), with 4000 mg/day particularly commonly used (505 patients in the remission stage, 357 patients in the active stage). Asacol® Tablets was administered most commonly at 3600 mg/day both in the remission stage and active stage (741 patients in the remission stage, 488 patients in the active stage). Salazopyrin® Tablets was administered most commonly at 3000 mg/day both in the remission stage and active stage (331 patients in the remission stage, 88 patients in the active stage) (Fig. 2).

Further analysis by stage/dosage and frequency revealed that Pentasa® Tablets was most frequently administered at 4000 mg/day in 2 divided doses (480 patients [21.0%] in the remission stage, 341 patients [46.6%] in the active stage), and Asacol® Tablets at 3600 mg/day in 3 divided doses (696 patients [46.4%] in the remission stage, 473 patients [67.3%] in the active stage), showing use of a high dose regardless of disease stage.

### Results of the overall patients included in the observational study of remission maintenance

**Fig. 3:**
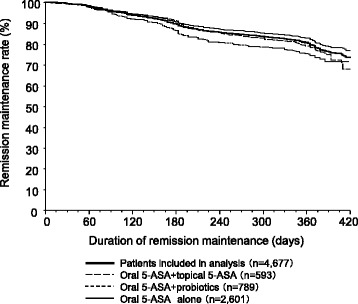
Cumulative remission maintenance rate

Among the 4677 patients included in the observational study of remission maintenance, the remission maintenance rate at week 52 was 80.2% (95% confidence interval [CI]: 79.0–81.3). The remission maintenance rate at week 52 by patients who were diagnosed within two years was 78.4%. The time-course of cumulative remission maintenance rate is shown in Fig. 3. The number of relapses was 0.2 ± 0.5 times/person-year, and the duration of remission maintenance was 311.6 ± 103.5 days.

### Remission maintenance rates by concomitant drugs, duration of disease

The remission maintenance rate at week 52 by drug was 82.1% for oral 5-ASA alone, 78.7% for oral 5-ASA + probiotics, and 77.1% for oral 5-ASA + topical 5-ASA. The time-course of cumulative remission maintenance rate for oral 5-ASA alone, oral 5-ASA + probiotics, and oral 5-ASA + topical 5-ASA is shown in Fig. 3.

The remission maintenance rate at week 52 by patient which was diagnosed within 2 years was 82.0% for oral 5-ASA alone, 73.1% for oral 5-ASA + probiotics, and 74.3% for oral 5-ASA + topical 5-ASA.

### Remission maintenance rates by drugs, by dosage and frequency, by the extent of lesion, and by clinical course in real life

**Fig. 4:**
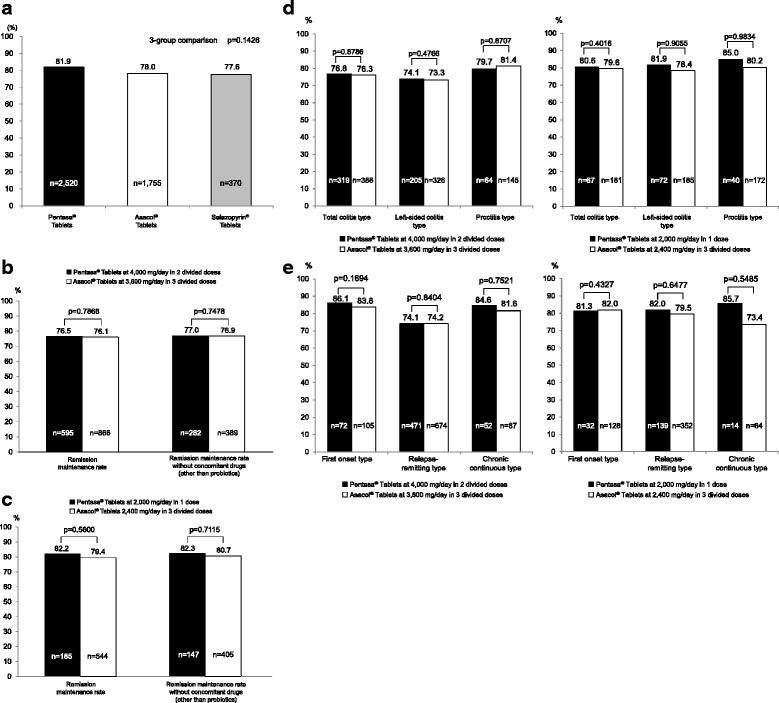
Remission maintenance rates. **a** Comparison by drug. Cochran-Mantel-Haenszel test. Adjustment factors: age (≤60 years, ≥61 years), classification by the extent of lesion, classification by clinical course, smoking habit, concomitant use of drugs for UC other than probiotics/cytapheresis therapy, duration of disease, duration of remission maintenance until enrollment (0 for patients in the active stage at enrollment). **b** Comparison between Pentasa® Tablets at 4000 mg/day in 2 divided doses and Asacol® Tablets at 3600 mg/day in 3 divided doses. Cochran-Mantel-Haenszel test. Adjustment factors: age (≤60 years, ≥61 years), classification by the extent of lesion, classification by clinical course, smoking habit, concomitant use of drugs for UC other than probiotics/cytapheresis therapy, duration of disease, duration of remission maintenance until enrollment (0 for patients in the active stage at enrollment). **c** Comparison between Pentasa® Tablets at 2000 mg/day in 1 dose and Asacol® Tablets 2400 mg/day in 3 divided doses. Cochran-Mantel-Haenszel test. Adjustment factors: age (≤60 years, ≥61 years), classification by the extent of lesion, classification by clinical course, smoking habit, concomitant use of drugs for UC other than probiotics/cytapheresis therapy, duration of disease, duration of remission maintenance until enrollment (0 for patients in the active stage at enrollment). **d** Comparison by the extent of lesion. Cochran-Mantel-Haenszel test. Adjustment factors: age (≤60 years, ≥61 years), classification by clinical course, smoking habit, concomitant use of drugs for UC other than probiotics/cytapheresis therapy, duration of disease, duration of remission maintenance until enrollment (0 for patients in the active stage at enrollment). **e** Comparison by clinical course. Cochran-Mantel-Haenszel test. Adjustment factors: age (≤60 years, ≥61 years), classification by the extent of lesion, smoking habit, concomitant use of drugs for UC other than probiotics/cytapheresis therapy, duration of disease, duration of remission maintenance until enrollment (0 for patients in the active stage at enrollment)

The remission maintenance rate at week 52 by drug was 81.9% for Pentasa® Tablets, 78.0% for Asacol® Tablets, and 77.6% for Salazopyrin® Tablets, and 3-group comparison after adjustment for adjustment factors showed no significant difference among the 3 groups (*p* = 0.1426, Fig. 4a).

The remission maintenance rate at week 52 by dosage and frequency did not significantly differ between Pentasa® Tablets at 4000 mg/day in 2 divided doses and Asacol® Tablets at 3600 mg/day in 3 divided doses which were bases of the case setting of the present study, or between Pentasa® Tablets at 2000 mg/day in 1 dose and Asacol® Tablets at 2400 mg/day in 3 divided doses, regardless of use of concomitant drugs (other than probiotics) (Fig. 4b, c).

Comparisons of the remission maintenance rate at week 52 by the extent of lesion and by clinical course also revealed no significant difference between Pentasa® Tablets at 4000 mg/day in 2 divided doses and Asacol® Tablets at 3600 mg/day in 3 divided doses, or between Pentasa® Tablets at 2000 mg/day in 1 dose and Asacol® Tablets at 2400 mg/day in 3 divided doses for either comparison (Fig. 4d, e).

### Remission maintenance rate by the presence or absence of dose reduction after remission had been induced in real life

**Fig. 5:**
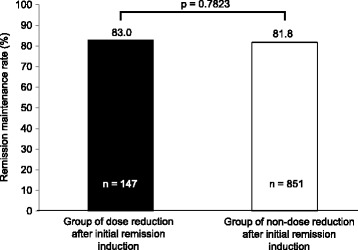
Remission Maintenance Rate by the Presence or Absence of Dose Reduction after Remission has been Induced. Cochran-Mantel-Haenszel test. Adjustment factors: age (≤60 years, ≥61 years), classification by the extent of lesion, classification by clinical course, smoking habit, concomitant use of drugs for UC other than probiotics/cytaphresis therapy, duration of disease, duration of remission maintenance until enrollment (0 for patients in the active stage at enrollment)

The number of cases in the active stage at enrollment that achieved remission induction by remission induction therapy and the number of cases in the remission stage at enrollment that had relapse during the observation period and then achieved remission induction by remission induction therapy totaled 1373. Of these cases, dose was not reduced after remission had been induced in 1181 cases (86.0%), supporting the general practice of not reducing dose immediately after remission induction. In the group receiving reduced dose after remission had been induced (dose reduction group) and the group receiving maintained dose (non-dose reduction group), the remission maintenance rate was 83.0 and 81.8%, respectively, showing almost the same values (*p* = 0.7823, Fig. 5).

### Factors affecting relapse

The following factors were identified to affect relapse, for patients with those factors at enrollment having a higher subsequent risk for relapse: left-sided colitis relative to the reference category of total colitis as the extent of lesion (hazard ratio [HR] = 1.159 [1.001–1.341], *p* = 0.0487); relapse-remitting type relative to the reference category of first onset type as disease stage (HR = 1.456 [1.198–1.770]; *p* = 0.0002); and bloody stool score 2 relative to the reference category of score 0 as symptom (HR 1.721 [1.126–2.628], *p* = 0.0120). In contrast, significantly persistent remission maintenance was noted in patients in whom duration of remission maintenance until enrollment was 12 to <24 months or ≥24 months relative to the reference category of <3 months (12 to <24 months: HR 0.600 [0.486–0.740], *p* < 0.0001]; ≥24 months: HR 0.352 [0.289–0.431], *p* < 0.0001) (Table 2).

**Table 2:** Factors Affecting Relapse

		Univariate analysis				Multivariate analysis^#1^		
		No. of patients	Hazard ratio	Confidence interval of hazard ratio	*P*-value for Wald test statistics	Hazard ratio	Confidence interval of hazard ratio	*P*-value for Wald test statistics
Age (years)	<20	4						
	20–30	538	0.778	0.109–5.563	*p* = 0.8028			
	31–40	1063	0.611	0.086–4.358	*p* = 0.6233			
	41–50	1114	0.545	0.076–3.887	*p* = 0.5450			
	51–60	869	0.491	0.069–3.504	*p* = 0.4777			
	61–70	713	0.414	0.058–2.966	*p* = 0.3802			
	≥71	373	0.336	0.046–2.435	*p* = 0.2807			
Sex	Male	2610						
	Female	2065	1.165	1.024–1.326	*p* = 0.0201	1.126	0.988–1.282	*p* = 0.0744
BMI	<18.5	485						
	18.5–<25.0	3354	0.883	0.718–1.087	*p* = 0.2411			
	≥25.0	801	0.837	0.653–1.074	*p* = 0.1630			
Duration of disease (months)	<12	344						
	12–<60	1424	0.808	0.631–1.034	*p* = 0.0900			
	60–<120	1238	0.738	0.573–0.950	*p* = 0.0186			
	≥120	1652	0.692	0.541–0.885	*p* = 0.0034			
Classification by the extent of lesion	Total colitistype	1942						
	Left-sided colitis type	1591	1.204	1.042–1.393	*p* = 0.0121	1.159	1.001–1.341	*p* = 0.0487
	Proctitis type	1043	0.910	0.762–1.086	*p* = 0.2937	0.957	0.800–1.144	*p* = 0.6271
	Other	99	0.773	0.461–1.295	*p* = 0.3282	0.858	0.512–1.439	*p* = 0.5618
Classification by linical course	First onset type	838						
	Relapse-remitting type	3427	1.608	1.325–1.952	*p* < 0.0001	1.456	1.198–1.770	*p* = 0.0002
	Chronic continuous type	410	1.379	1.024–1.859	*p* = 0.0344	1.133	0.837–1.534	*p* = 0.4175
Smoking habit	Yes	1222						
	No	3442	1.036	0.893–1.201	*p* = 0.6421			
Drinking habit	No	2631						
	≤1 day/week	992	1.060	0.900–1.247	*p* = 0.4865			
	2–5 days/week	595	1.058	0.867–1.289	*p* = 0.5797			
	≥6 days/week	443	1.050	0.842–1.309	*p* = 0.6670			
Complications	Yes	965						
	No	3712	1.094	0.930–1.286	*p* = 0.2788			
Employment status	Student	108						
	Part-time worker	534	0.751	0.497–1.135	*p* = 0.1740			
	Full-time worker	2632	0.705	0.482–1.031	*p* = 0.0714			
	Other	1401	0.626	0.424–0.925	*p* = 0.0186			
Concomitant use of infliximab/adalimumab	Yes	129						
	No	4548	1.319	0.847–2.054	*p* = 0.2205			
Concomitant use of tacrolimus	Yes	26						
	No	4651	0.722	0.300–1.738	*p* = 0.4674			
Concomitant use of immunomodulator	Yes	602						
	No	4075	0.874	0.727–1.051	*p* = 0.1517			
Concomitant use of probiotics	Yes	789						
	No	3888	0.919	0.777–1.086	*p* = 0.3208			
Concomitant use of 5-ASA enema/suppository	Yes	594						
	No	4083	0.757	0.631–0.909	*p* = 0.0028			
Drug adherence at enrollment	<80%	705						
	≥80%	3941	1.132	0.939–1.364	*p* = 0.1926			
Type of oral 5-ASA products at enrollment	Pentasa® Tablets	2528						
	Asacol® Tablets	1744	1.301	1.136–1.490	*p* = 0.0001			
	Salazopyrin® Tablets	368	1.273	1.007–1.608	*p* = 0.0435			
	Other (generic)	16	0.000	0.000 > 999.999	*p* = 0.9351			
	Concomitant use (oral products)	22	1.092	0.408–2.921	*p* = 0.8615			
Daily dose of oral 5-ASA products at enrollment (mg)	<1500	287						
	≥1500–<2500	1982	1.190	0.864–1.640	*p* = 0.2874			
	≥2500	2408	1.780	1.301–2.436	*p* = 0.0003			
Duration of remission maintenance prior to enrollment^#2^ (months)	<3	1074						
	≥3–<12	1013	0.854	0.721–1.011	*p* = 0.0662	0.928	0.772–1.115	*p* = 0.4236
	≥12–<24	824	0.542	0.445–0.661	*p* < 0.0001	0.600	0.486–0.740	*p* < 0.0001
	≥24	1703	0.316	0.263–0.380	*p* < 0.0001	0.352	0.289–0.431	*p* < 0.0001
Stool frequency score at enrollment	0	3539						
	1	874	1.331	1.132–1.564	*p* = 0.0005	1.129	0.952–1.339	*p* = 0.1642
	2	195	1.788	1.351–2.366	*p* < 0.0001	1.334	0.986–1.806	*p* = 0.0618
	3	69	0.882	0.439–1.772	*p* = 0.7243	0.618	0.303–1.262	*p* = 0.1864
Bloody stool score at enrollment	0	4159						
	1	413	1.693	1.371–2.090	*p* < 0.0001	1.249	0.988–1.578	*p* = 0.0631
	2	98	2.712	1.845–3.987	*p* < 0.0001	1.721	1.126–2.628	*p* = 0.0120
	3	7	0.000	0.000–>999.999	*p* = 0.9476	0.000	0.000–>999.999	*p* = 0.9446
PGA score at enrollment	0	3830						
	1	770	1.564	1.324–1.847	*p* < 0.0001			
	2	76	1.212	0.668–2.200	*p* = 0.5275			
	3	1	0.001	0.000–>999.999	*p* = 0.9535			

#1 Factors affecting relapse in terms of the duration until relapse were investigated using a Cox regression model (multivariate) with the items having *p* < 0.05 in univariate analysis as explanatory variables

#2 Duration of remission maintenance was counted as 0 month for the patients in active stage at enrollment

### Drug adherence

Drug adherence was 90.5% ± 14.2% (*n* = 5648) at enrollment, 90.8% ± 13.8% (*n* = 5, 332) at week 26, and 91.0% ± 13.8% (*n* = 4984) at week 52, confirming extremely favorable adherence. The drug adherence by dosage and frequency at enrollment was 91.2% ± 13.5% (*n* = 409) for one dose/day, 90.8% ± 14.6% (*n* = 2324) for 2 divided doses/day, 90.2% ± 14.0% (*n* = 2848) for 3 divided doses/day, and 86.6% ± 14.2% (*n* = 65) for 4 divided doses/day, showing favorable adherence regardless of dosage and frequency.

## Discussion

In the present study, 5695 patients were enrolled from 379 sites in Japan. It was confirmed that the distributions of the residential area classification, age, and sex of enrolled patients were almost consistent with those of the Report on Public Health Frequency and Services FY2012 by the Ministry of Health, Labour and Welfare [11]. Compared with the Results of Tabulation of Personal Health Records FY2007 [12], enrolled patients showed a distribution by classification of clinical course with a slightly higher proportion of the relapse-remitting type and a similar distribution by disease type classification based on the extent of lesion. Furthermore, as compared with the CARE study, conducted in Germany, enrolled patients had longer duration of disease regardless of the classification of disease stage and higher percentage of total colitis in disease type classification based on the extent of lesion; however, the tendency was generally similar [13]. Accordingly, the study population was considered to be representative of the UC patient populations both in Japan and overseas.

It was as follows that it was thought for reasons of drop off treatment or follow up.

(1) No visits after enrollment: in the remission stage, self-interruption of the treatment with remission continuing and custom having occurred, in the active stage, self-interruption by the symptom improvement, the hospital transfer by a symptom not being improved, (2) Remission induced at week 26 and no subsequent data available: self-interruption by the symptom improvement, (3) Oral 5-ASA products discontinued before week 26: changing to treatment except the oral 5-ASA product prescription.

Among 4677 patients included in the analysis population (patients in the remission stage at enrollment and those in the active stage at enrollment who had achieved remission induction), remission maintenance rate at week 52 was 80.2%. A remission maintenance rate of oral 5-ASA alone was slightly high as a result of real life. It was thought that these results had more oral 5-ASA alone the calm patients with symptom.

Also, remission maintenance rate of real life, oral 5-ASA alone, oral 5-ASA + probiotics, oral 5-ASA + topical 5-ASA were higher than the patients which were diagnosed within two years. It was thought to be many ratios of patients that the activity was higher in the patients which were as diagnosed within two years.

By drug, the remission maintenance rate was 81.9% (*n* = 2520) for Pentasa®, 78.0% (*n* = 1755) for Asacol® Tablets, and 77.6% (*n* = 370) for Salazopyrin® Tablets, showing high values for all the drugs. The absence of significant difference in remission maintenance rate among the 3 groups suggests that the type of drug hardly affects efficacy in clinical practice despite the difference in release profile. The lack of significant difference in remission maintenance rate in the comparisons between Pentasa® Tablets at 2000 mg/day in one dose and Asacol® Tablets at 2400 mg/day in 3 divided doses, and between Pentasa® Tablets at 4000 mg/day in 2 divided doses and Asacol® Tablets at 3600 mg/day in 3 divided doses further supported the discussion above.

On the other hand, it was confirmed that patients with remission maintained for 12 months or longer prior to enrollment have a reduced subsequent risk for relapse than those with remission maintained for less than 12 months. Green, et al. reported that annual relapse rate was 35% in patients who newly achieved remission induction after enrollment, compared with 21% in those who had maintained remission since before enrollment [14]. The present study also identified relationship between the duration of remission maintenance and risk for relapse and demonstrated that treatment that enables remission maintenance for at least 12 months leads to a reduction in subsequent risk for relapse. This is of extreme significance in that it determined the approximate desirable duration of remission induction. The reason that female patients tended to be relapse did not unknown. Bitton A, et al. reported that multivariate Cox regression analysis retained greater number of prior relapses in women as predictors of shorter time to clinical relapse. However, there was no significant effect of medication use on the strong interaction between gender (female) and number of prior relapses [15].

The results of the present study suggested that 12-month or longer continued use of the dose that successfully introduces remission would be an approach to reducing a risk for relapse in treatment with oral 5-ASA products. The comparison between the group receiving reduced dose after remission had been first induced (dose reduction group) and the group receiving maintained dose (non-dose reduction group), however, failed to show definite difference in remission maintenance rate. This is explained as follows: In the present study, the duration of observation was set at 52 weeks from the time of enrollment; therefore, for example, for patients in the active stage at enrollment, the duration required for remission induction was to be included in the duration of observation, resulting in the duration of remission maintenance shorter than 52 weeks. Nevertheless, if in the remission stage at week 52, the cases were judged to have maintained remission, resulting in a failure to demonstrate the difference between the presence and absence of dose reduction after remission had been induced. And we did not conduct sub analysis of endoscopic evaluation. A future long-term observational study starting from the time of remission induction involving endoscopic evaluation will reveal a difference in remission maintenance rate between the presence and absence of dose reduction.

The study of possible relationship between relapse and adherence in remission-stage UC patients by Kane, et al. reported that all of the patients with relapse at month 6 of follow-up (12%) and 68% of patients with relapse at month 12 months were non-adherent patients (adherence < 80%) [16]. In addition, Kawakami, et al. reported twice higher risk for relapse in non-adherent patients than in adherent patients [17], suggesting relationship between reduced adherence and increased risk for relapse. Furthermore, as for the reasons for drug adherence, Kane, et al. cited skipped doses (50%), the large number of tablets (30%), and no need felt (20%) [16], and Hawthorne, et al. reported involvement of frequent dosing (3 times daily) [18]. Meanwhile, the survey by Kawakami, et al. reported extremely high adherence in Japanese UC patients [17], and the study by Watanabe, et al. did not provide evidence for the effect of the number of doses [19]. As the reason for high adherence in Japanese patients, Japanese temperament and direct consultation with IBD expert as well as lack of financial concerns thanks to financial support system for UC patients (Research on Measures for Intractable Diseases) have been cited [17].

The present study confirmed high remission maintenance rate at week 52, which is considered to be attributable to Japanese-unique favorable drug adherence. It would therefore be fundamental to UC treatment to fully convince patients of the importance of drug adherence for reduction in relapse risk even in the asymptomatic remission stage, thereby pursuing enhancement of adherence.

The data of the concomitant medication describe the drug which they used together at enrollment.

## Conclusions

The present study demonstrated consistently high drug adherence and favorable remission-maintaining effect of real life results in using oral 5-ASA products for maintaining mild to moderate UC patients in Japan. This study also suggested the importance of continuing remission maintenance for at least 12 months for a reduction in a risk for relapse.

## Additional files

Additional file 1: A questionnaire on adherence to medication of the oral 5-ASA products. (DOC 47 kb)

Additional file 2: The name of committee. (XLS 50 kb)

## Abbreviations

5-ASA: 5-aminosalicylic acid
6-MP: 6-Mercaptopurine
BMI: Body Mass Index
CI: Confidence interval
HR: Hazard ratio
IBD: Inflammatory bowel disease
PGA: Physician’s global assessment
SD: Standard deviation
UC: Ulcerative colitis
UCDAI: UC disease activity index
VAS: Visual analog scale.
