# Supplementary material for: Real life results in using 5-ASA for maintaining mild to moderate UC patients in Japan, a multi-center study, OPTIMUM Study
Source: BMC Gastroenterol. 2017 Apr 4;17:47. doi: 10.1186/s12876-017-0604-y (PMC5385245; doi:10.1186/s12876-017-0604-y)
Supplement: Supplementary file 1 — A questionnaire on adherence to medication of the oral 5-ASA products. (DOC 47 kb) [file 12876_2017_604_MOESM1_ESM.doc]

Additional file 1.

**‘A questionnaire on adherence to medication of the oral 5-ASA products’**

On adherence to medication of the oral 5-ASA products which is prescribed (is taken), please answer the questionnaire (visual analog scale) in reference to sample 1,2. The physician may not look at the questionnaire after you replied it.

**visual analog scale**

　　　　　　0％　　　　　　　　　　　　　　　　　　　　　　　　　100％

**Sample.1：The case which was able to take 60% of the oral 5-ASA products**

　　　　　　0％　　　　　　　　　　　　　　　　　　　　　　　　　100％

**Sample.2：The case which was able to take 90% of the oral 5-ASA products**

0％　　　　　　　　　　　　　　　　　　　　　　　　　100％

After you replied the questionnaire on adherence to medication of the oral 5-ASA products, please put it in the envelope and seal it, and hand it to the physician or the staff. Thank you for your cooperation.
